# Three-Dimensional Accuracy of Digitally Planned Orthodontic Tooth Movement in a Fully Customized Self-Ligating Lingual System

**DOI:** 10.3390/bioengineering13010094

**Published:** 2026-01-14

**Authors:** Arda Arısan, Tülin Taner

**Affiliations:** 1 Independent Researcher, Ankara 06510, Turkey; 2 Department of Orthodontics, Faculty of Dentistry, Hacettepe University, Ankara 06230, Turkey

**Keywords:** customized lingual orthodontic system, biomechanical accuracy, digital treatment planning, three-dimensional analysis

## Abstract

**Background**: Lingual orthodontic systems have recently advanced with the introduction of fully customized CAD/CAM-based designs featuring self-ligating (SL) mechanisms. This study aimed to evaluate the three-dimensional accuracy of a customized SL lingual system in reproducing digitally planned tooth positions. **Methods**: A total of 280 teeth were analyzed following treatment with a fully customized self-ligating lingual system (Harmony^®^, Aso International Inc., Tokyo, Japan). Digital models obtained before treatment (T0), from the setup (TS), and after treatment (T1) were superimposed using a best fit algorithm in GOM Inspect. Tooth movements were quantified across seven biomechanically relevant parameters including tip, torque, rotation, buccolingual, mesiodistal, vertical, and overall displacement. Predicted and achieved movements were compared using paired *t* tests and Bland–Altman analysis. **Results**: The fully customized SL lingual appliance achieved an overall dentition accuracy of 92.1%. Mean accuracy for linear tooth movements was 94.5% ± 2.1% in the maxilla and 93.8% ± 2.5% in the mandible. For angular movements, mean accuracy was 90.8% ± 3.4% in the maxilla and 89.3% ± 3.9% in the mandible. The highest precision was observed in anterior teeth for mesiodistal (96.2%) and buccolingual (95.8%) movements, whereas the lowest accuracy occurred in rotational movements of the posterior segments (87.1%). No statistically significant differences were found between predicted and achieved movements for most parameters (*p* > 0.05). **Conclusions**: The fully customized SL lingual orthodontic system demonstrated high accuracy in reproducing digitally planned tooth movements, particularly in the anterior segments. Although accuracy was slightly lower in the posterior regions, the overall outcomes remained mechanically and clinically acceptable across all evaluated dimensions.

## 1. Introduction

Lingual orthodontics has increasingly incorporated fully customized CAD/CAM-based bracket systems that allow precise adaptation to individual dental morphology and clinical requirements [1]. These computer-designed and laboratory-manufactured appliances enable individualized bracket positioning and accurate control of archwire geometry, thereby optimizing tooth movement and overall biomechanics [2]. Alongside this development, the introduction of self-ligating (SL) designs has supported more efficient and comfortable treatment protocols [3]. Clinically, SL configurations may reduce chair time and simplify archwire engagement [4], while also meeting the high esthetic expectations of adult patients seeking discreet orthodontic treatment [5].

The accuracy of reproducing digitally planned tooth positions is a key indicator of the clinical performance of contemporary orthodontic appliances [6] and is commonly assessed through three-dimensional comparisons between the virtual setup and the final treatment outcome [7]. Several studies have specifically evaluated the accuracy with which customized systems transfer digitally planned bracket positions to the dentition during the indirect bonding process [8,9,10]. Similar analyses of labially placed customized systems have demonstrated that bracket positioning accuracy may vary depending on the bonding protocol, bracket design, and manufacturing workflow [11,12].

Clear aligner therapy has gained widespread acceptance due to its favorable esthetics and high patient acceptance among both adult and adolescent patients [13,14]. Consequently, numerous studies have investigated the accuracy with which aligners reproduce digitally planned tooth movements and achieve predictable clinical outcomes [15,16,17,18,19,20]. While predictable results have been reported for simple alignment and minor space closure, reduced accuracy persists for rotations, intrusions, and torque movements when compared with fixed appliances [20,21,22,23]. These limitations become particularly evident when treatment objectives involve complex movements or significant vertical discrepancies.

For patients who prioritize esthetics under challenging treatment conditions, lingual orthodontic appliances represent a reliable alternative. Previous studies have demonstrated that their biomechanical efficiency is comparable to that of conventional labial systems, enabling precise tooth movement while maintaining effective esthetic concealment [24,25,26,27,28]. Despite these advantages, the amount of clinical evidence available on fully customized SL lingual systems remains relatively limited. Further well-designed studies are required to confirm their accuracy, long term stability, and overall clinical performance in complex treatment scenarios.

The aim of this study was to evaluate the accuracy with which a fully customized SL lingual orthodontic appliance (Harmony^®^, Aso International Inc., Tokyo, Japan) reproduces the planned digital treatment setup using three-dimensional superimposition and quantitative analysis.

## 2. Materials and Methods

### 2.1. Study Design and Ethical Approval

This retrospective study was conducted in accordance with the Declaration of Helsinki and was approved by the Clinical Research Ethics Committee of Hacettepe University (Decision No: KA-15016). Additionally, compliance certification was obtained from the Turkish Medicines and Medical Devices Agency (TMMDA), approval number 71146310 [2016-CE-004]. All participants provided written informed consent prior to inclusion. All patients were treated at the Hacettepe University Faculty of Dentistry, Department of Orthodontics.

### 2.2. Participants and Eligibility Criteria

15 adults who had completed skeletal growth and requested lingual orthodontic treatment for esthetic reasons were screened. Inclusion criteria were Class I malocclusion with stable habitual occlusion, skeletal symmetry, a mesofacial growth pattern (FMA: 25° ± 4), and mild to moderate arch length deficiency in both arches (2–5 mm). Exclusion criteria included a need for orthognathic surgery or skeletal anchorage, skeletal anomalies, an unreproducible maximum intercuspation, or incomplete records. 10 consecutive patients (7 females, 3 males; mean age, 27.3 ± 6.9 years; range, 18–38 years) met the criteria, providing 20 dental arches and 280 teeth for three-dimensional evaluation.

### 2.3. Data Collection and Digital Setup

**Figure 1:**
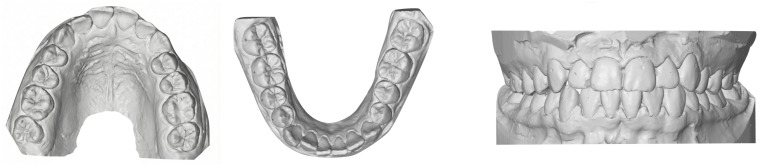
Sample STL files from the T0 time point. The maxillary arch (**left**), mandibular arch (**center**), and occlusal bite registration (**right**) are shown separately to illustrate the three digital records generated for each patient.

Standard orthodontic records were obtained at T0 (pretreatment) and T1 (posttreatment). Digital models were generated using a 7Series desktop optical scanner (Dental Wings, Montreal, QC, Canada) at 15 μm resolution. The treatment setup (TS) was created by the Harmony Technical Center (ASO International Inc., Japan) and delivered in STL format. For each time point (T0, TS, T1), three STL files were generated per patient: maxillary arch, mandibular arch, and occlusal bite registration (Figure 1).

### 2.4. Orthodontic Treatment Protocol

**Figure 2:**
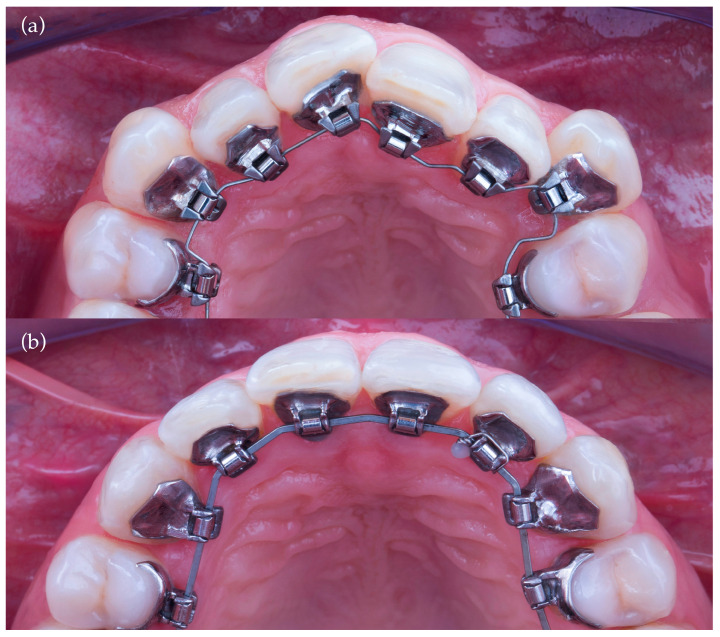
Occlusal views of Harmony lingual brackets at two different stages of treatment. (**a**) Initial stage with a 0.014-inch NiTi archwire. (**b**) Final stage with a 0.018 × 0.025-inch NiTi archwire.

After approval of the setup, fully customized self-ligating lingual brackets and transfer trays were fabricated using CAD/CAM technology. Indirect bonding was performed on etched lingual enamel surfaces (37% phosphoric acid) using a dual-cure resin cement (Maxcem Elite, Kerr, Orange, CA, USA). Brackets were light cured according to the manufacturer’s recommendations, with additional curing performed after tray removal. The archwire sequence consisted of 0.014-inch NiTi, 0.016 × 0.016-inch NiTi, 0.016 × 0.022-inch NiTi, and 0.018 × 0.025-inch NiTi. Interproximal reduction was performed according to the digital plan. Treatment was completed after achieving Class I canine and molar relationships and ideal overbite (0–3 mm) and overjet (2–3 mm), based on the American Board of Orthodontics Objective Grading System thresholds [29]. A fixed lingual retainer (Bond-A-Braid, Reliance Orthodontic Products, Itasca, IL, USA) was bonded from canine to canine in both arches and used together with an Essix retainer. The progressive changes in archwire dimensions and bracket configurations during treatment are illustrated in Figure 2.

### 2.5. Digital Model Processing and Superimposition

STL models (T0, TS, T1) were imported into GOM Inspect (GOM GmbH, Braunschweig, Germany). The T0 maxillary model was oriented to the local coordinate system using the incisive papilla as the origin. Maxillary superimposition was performed using initial three-point registration on palatal rugae followed by best-fit surface alignment, selected due to their documented stability [30,31]. The setup (TS) and posttreatment (T1) models were aligned to T0 using the same protocol.

**Figure 3:**
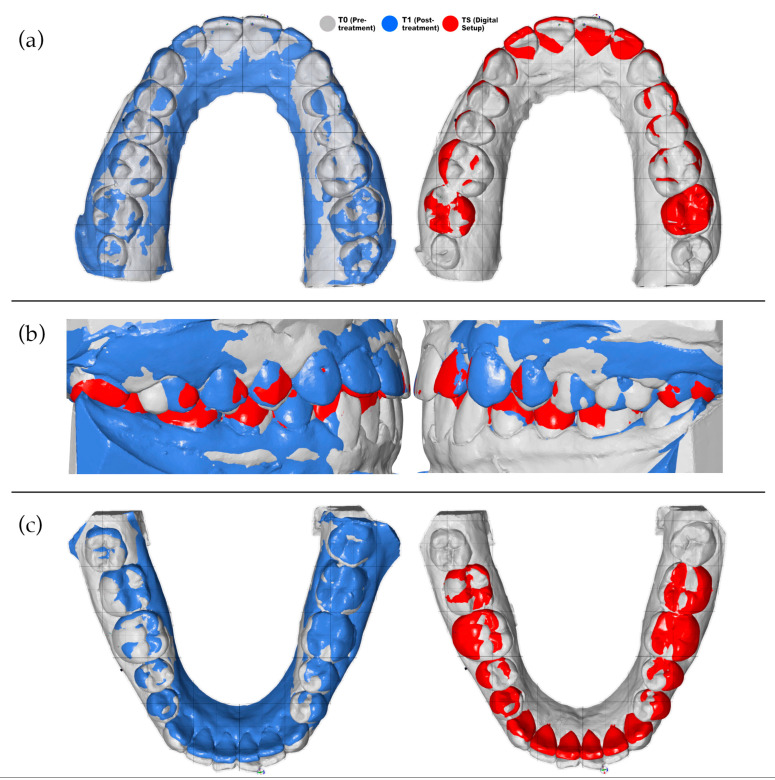
Superimposition of digital models. (**a**) Maxillary superimpositions demonstrating the T0–T1 and T0–TS alignments. (**b**) Bite registration superimposition illustrating the combined T0–T1–TS alignment. (**c**) Mandibular superimpositions showing the T0–T1 and T0–TS alignments. Color coding: T0 (gray), T1 (blue), TS (red).

The mandibular models were registered with the help of the bite scan. The bite scan was aligned to the maxillary models, and its maxillary portion was then removed, leaving the mandibular segment positioned in the same coordinate system. Registration was refined by identifying lingual cortical bone contours and applying best-fit surface alignment. Figure 3 shows color-coded superimpositions illustrating the T0–T1 and T0–TS alignments, as well as the combined T0–T1–TS alignment obtained from bite registration.

### 2.6. Tooth Movement Measurements

**Figure 4:**
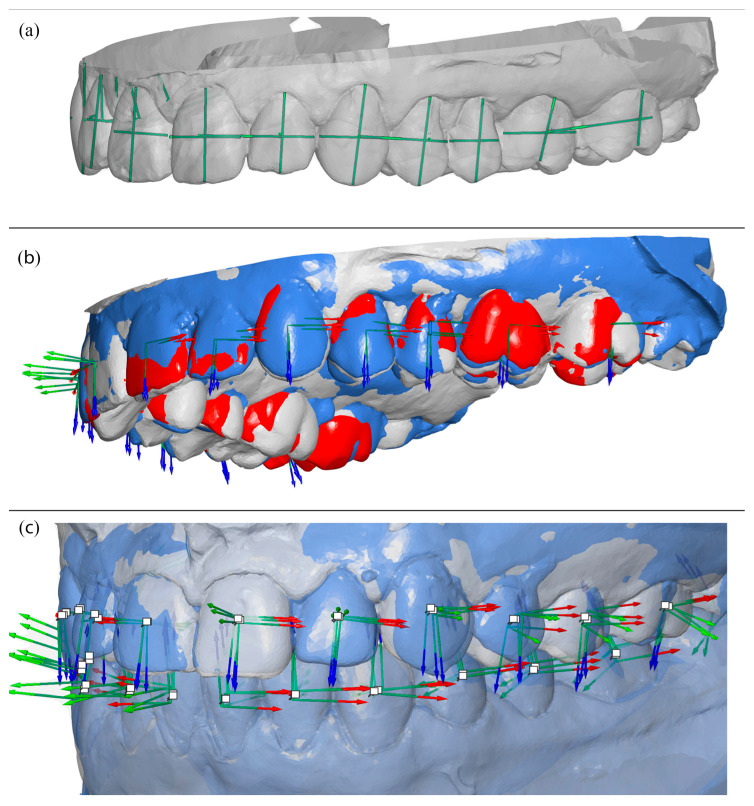
Visualization of reference points, reference planes, and model superimpositions: (**a**) reference points and planes used for measurements; (**b**) demonstrative maxillary superimposition of pretreatment (T0), posttreatment (T1), and digital setup (TS) models; (**c**) demonstrative overall superimposition of pretreatment (T0) and posttreatment (T1) models. Colored arrows indicate the direction and magnitude of planned and achieved tooth movements along the defined coordinate axes.

After model alignment, reference points and axes were established for each tooth in all models. The facial axis point was used as the reference point, with the facial axis of the clinical crown defined as the tooth axis. A horizontal axis and a vertical axis were also assigned to standardize angular measurements. These reference points and planes served as the basis for calculating angular and linear tooth movements, as illustrated in Figure 4.

**Figure 5:**
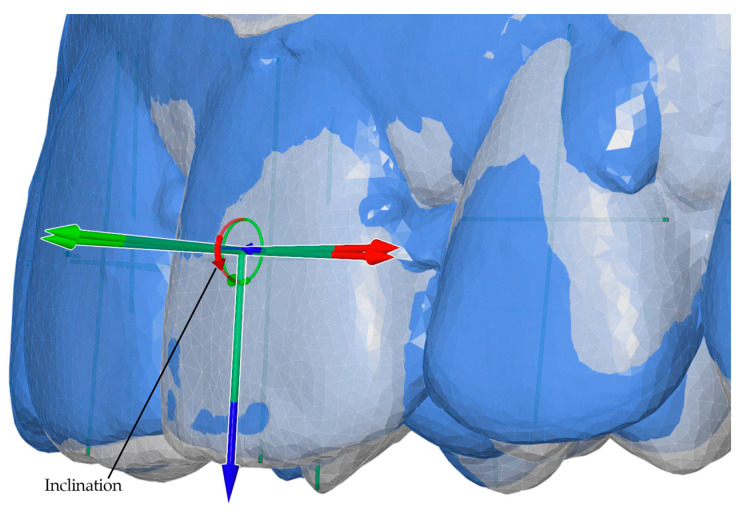
Angular measurement of inclination (Phi, X°) for anterior teeth, illustrated for the T0–T1 interval (pretreatment to posttreatment). The red circular arrow represents inclination around the X-axis.

**Figure 6:**
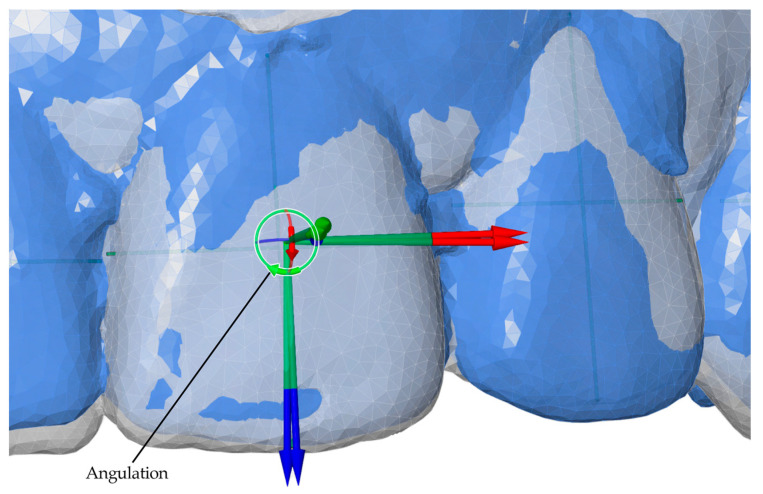
Angular measurement of angulation (Theta, Y°) for anterior teeth, illustrated for the T0–T1 interval (pretreatment to posttreatment). The green circular arrow represents angulation around the Y-axis.

**Figure 7:**
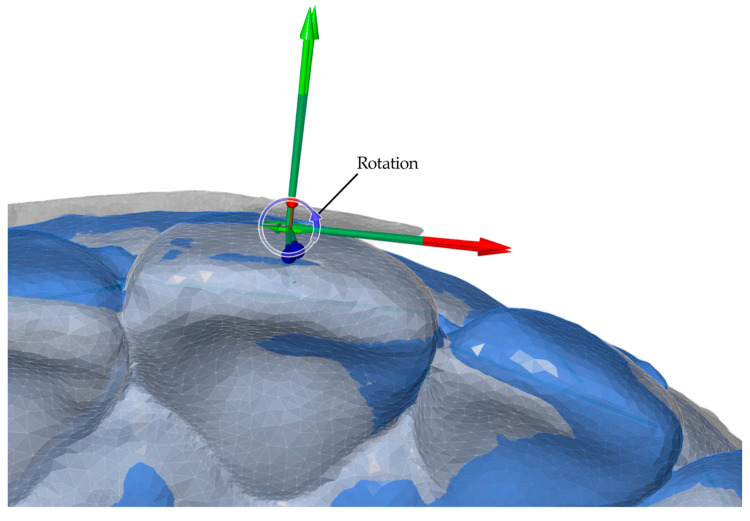
Angular measurement of rotation (Psi, Z°) for teeth, illustrated for the T0–T1 interval (pretreatment to posttreatment). The blue circular arrow represents rotation around the Z-axis.

**Figure 8:**
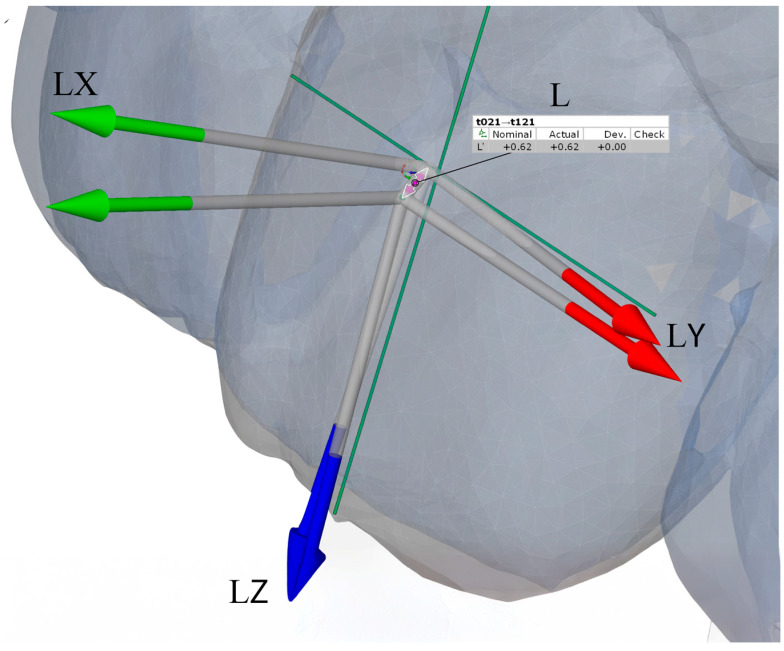
Illustration of linear measurements of 3D displacement for anterior teeth: buccolingual (LX, green arrows), mesiodistal (LY, red arrows), and vertical (LZ, blue arrows) components, together with the overall translational displacement (L, purple arrows), calculated as the vector magnitude of LX, LY, and LZ.

Angular measurements between reference planes and linear measurements between reference points were performed for two intervals: T0–T1 (pretreatment to posttreatment) and T0–TS (pretreatment to digital setup). Movements were evaluated along seven parameters, including three angular (Phi, Theta, Psi) and four linear (L, LX, LY, LZ) components. Linear movements were measured in millimeters, while angular movements were measured in degrees. Detailed definitions and axis-specific interpretations for anterior and posterior teeth are summarized in Table 1. Representative examples of these measurement parameters are presented in Figure 5, Figure 6, Figure 7 and Figure 8.

### 2.7. Statistical Analysis

A priori power analysis was performed using G*Power 3.1 software (Heinrich Heine University, Düsseldorf, Germany) to estimate the required sample size for paired *t*-test analysis. Based on previous orthodontic accuracy studies [32], assuming a medium effect size (Cohen’s d = 0.5), an alpha level of 0.05, and a desired power of 0.80, a minimum of 34 paired measurements was required.

To reduce intra-subject variability, 280 teeth were grouped into 140 symmetrical pairs according to the FDI tooth numbering system, with each pair serving as one analytical unit. Seven movement parameters were analyzed, including three angular (torque, angulation, and rotation) and four linear (L, LX, LY, and LZ) displacements. Analyses were performed at the paired-tooth unit level; potential within-subject clustering is acknowledged as an inherent limitation. Accuracy was calculated using the formula described by Kravitz et al. [33]: Accuracy (%) = 100 − (|predicted − achieved|/|predicted| × 100).

All statistical analyses were performed using IBM SPSS Statistics 20.0 (IBM Corp., Armonk, NY, USA). Predicted (T0–TS) and achieved (T0–T1) tooth movements were compared using paired sample *t* tests. Agreement was evaluated using Bland–Altman analysis, and reliability was assessed using intraclass correlation coefficients (ICCs). Statistical significance was set at *p* < 0.05.

## 3. Results

### 3.1. Quantitative Movement Analysis

Table 2a,b and Table 3a,b present detailed measurements of planned (T0–TS) versus achieved (T0–T1) tooth movements across all spatial parameters. The results show systematic variations in precision between anatomical regions and movement types, with linear movements generally demonstrating higher accuracy than angular movements.

**Table 2:** (**a**) Predicted versus achieved tooth movements in the maxillary anterior region, showing linear and angular parameters with accuracy values and statistical significance. (**b**) Predicted versus achieved tooth movements in the maxillary posterior region, showing linear and angular parameters with accuracy values and statistical significance.

**(a)**								
		**Total**	**Buccolingual**	**Mesiodistal**	**Vertical**	**Inclination**	**Angulation**	**Rotation**
		**L**	**LX**	**LY**	**LZ**	**Phi (X)**	**Theta (Y)**	**Psi (Z)**
11,21	T0–TS	1.94 ± 1.42	0.94 ± 1	0.58 ± 0.29	0.74 ± 0.69	5.77 ± 2.44	3.01 ± 2.37	5.41 ± 3.24
	T0–T1	1.81 ± 1.76	0.92 ± 0.85	0.5 ± 0.39	0.56 ± 0.37	5.01 ± 2.16	2.82 ± 2.32	5.31 ± 3.3
	*p*	0.569	0.826	0.431	0.227	0.111	0.354	0.796
	Accuracy %	93.3	97.9	86.2	75.7	86.8	93.7	98.2
12,22	T0–TS	2.56 ± 1.63	1.51 ± 1.35	1.11 ± 0.8	0.82 ± 0.44	5.57 ± 3.59	3.98 ± 2.36	7.17 ± 1.74
	T0–T1	2.37 ± 1.54	1.38 ± 1.27	1.06 ± 0.86	0.81 ± 0.53	5.18 ± 3.79	4.04 ± 2.07	6.57 ± 1.87
	*p*	0.108	0.184	0.623	0.925	0.357	0.925	0.110
	Accuracy %	92.6	91.4	95.5	98.8	93.0	99.0	91.6
13,23	T0–TS	1.62 ± 0.76	0.92 ± 0.72	0.59 ± 0.46	0.98 ± 0.47	5.82 ± 2.92	5.16 ± 2.78	5.03 ± 1.91
	T0–T1	1.65 ± 0.6	0.87 ± 0.5	0.7 ± 0.4	0.85 ± 0.58	4.74 ± 2.41	5.3 ± 2.04	4.89 ± 1.75
	*p*	0.710	0.692	0.265	0.380	0.089	0.768	0.539
	Accuracy %	99.0	94.6	99.0	86.7	81.4	99.0	97.2
(**b**)								
		**Total**	**Buccolingual**	**Mesiodistal**	**Vertical**	**Inclination**	**Angulation**	**Rotation**
		**L**	**LY**	**LX**	**LZ**	**Theta (Y)**	**Phi (X)**	**Psi (Z)**
14,24	T0–TS	1.72 ± 0.61	0.91 ± 0.37	0.81 ± 0.69	0.91 ± 0.37	4.66 ± 3.65	5.41 ± 4.04	4.77 ± 1.97
	T0–T1	1.57 ± 0.57	0.74 ± 0.28	0.89 ± 0.57	0.88 ± 0.35	4.94 ± 4.02	5.54 ± 3.46	5.05 ± 1.96
	*p*	0.104	0.073	0.521	0.735	0.643	0.755	0.391
	Accuracy %	91.3	81.3	99.0	96.7	99.0	99.0	99.0
15,25	T0-TS	1.81 ± 0.72	0.71 ± 0.38	0.95 ± 0.64	1.13 ± 0.46	3.15 ± 2.07	5.85 ± 3.16	4.58 ± 1.33
	T0-T1	1.62 ± 0.62	0.58 ± 0.33	0.96 ± 0.46	0.96 ± 0.47	3.32 ± 1.94	4.54 ± 2.31	4.02 ± 1.94
	*p*	0.102	0.041 *	0.974	0.045 *	0.707	0.045 *	0.329
	Accuracy %	89.5	81.7 *	99.0	85.0 *	99.0	77.6 *	87.8
16,26	T0–TS	1.75 ± 0.69	0.69 ± 0.39	1.13 ± 0.66	0.77 ± 0.53	3.69 ± 1.58	3.57 ± 1.47	5.15 ± 1.88
	T0–T1	1.49 ± 0.66	0.47 ± 0.23	1.08 ± 0.54	0.66 ± 0.52	3.69 ± 1.83	2.61 ± 1.34	4.38 ± 2.34
	*p*	0.039 *	0.049 *	0.718	0.099	0.995	0.092	0.027 *
	Accuracy %	85.1 *	68.1 *	95.6	85.7	99.0	73.1	85.0 *
17,27	T0–TS	2.51 ± 2.03	0.76 ± 0.55	1.13 ± 0.72	1.12 ± 0.76	8.63 ± 4.35	6.96 ± 3.57	6.04 ± 3.02
	T0–T1	2.28 ± 1.65	0.6 ± 0.52	1.32 ± 0.96	0.63 ± 0.58	6.74 ± 4.24	6.39 ± 3.05	5.27 ± 2.57
	*p*	0.225	0.231	0.241	0.027 *	0.006 *	0.562	0.227
	Accuracy %	90.8	78.9	99.0	56.3 *	78.1 *	91.8	87.2

* *p* < 0.05 indicates statistically significant difference between predicted (T0–TS) and achieved (T0–T1) movements.

**Table 3:** (**a**) Predicted versus achieved tooth movements in the mandibular anterior region, showing linear and angular parameters with accuracy values and statistical significance. (**b**) Predicted versus achieved tooth movements in the mandibular posterior region, showing linear and angular parameters with accuracy values and statistical significance.

**(a)**								
		**Total**	**Buccolingual**	**Mesiodistal**	**Vertical**	**Inclination**	**Angulation**	**Rotation**
		**L**	**LX**	**LY**	**LZ**	**Phi (X)**	**Theta (Y)**	**Psi (Z)**
31,41	T0–TS	2.24 ± 0.63	0.76 ± 0.73	0.91 ± 0.56	1.56 ± 0.55	6.98 ± 4.23	4.08 ± 2.24	7.99 ± 4.09
	T0–T1	2.28 ± 0.9	1.02 ± 1.18	0.94 ± 0.41	1.27 ± 0.63	6.14 ± 3.38	4.35 ± 3.06	8.1 ± 4.1
	*p*	0.743	0.158	0.806	0.171	0.154	0.577	0.883
	Accuracy %	99.0	99.0	99.0	81.4	88.0	99.0	99.0
32,42	T0–TS	2.06 ± 0.81	1 ± 0.72	0.92 ± 0.69	1.36 ± 0.54	4.7 ± 2.79	5.16 ± 4	6.71 ± 4.62
	T0–T1	2.06 ± 0.8	1.12 ± 1.02	0.88 ± 0.54	1.27 ± 0.46	4.53 ± 2.28	5.49 ± 3.75	7.34 ± 4.71
	*p*	0.988	0.472	0.706	0.205	0.543	0.545	0.334
	Accuracy %	99.0	99.0	95.7	93.4	96.4	99.0	99.0
33,43	T0–TS	2.34 ± 0.85	1.38 ± 0.86	1.2 ± 0.75	1.31 ± 0.72	5.48 ± 2.74	5.68 ± 2.34	6.87 ± 3.18
	T0–T1	2.52 ± 0.92	1.51 ± 1.16	1.19 ± 0.64	1.22 ± 0.4	4.99 ± 2.55	5.55 ± 2.24	7.21 ± 2.87
	*p*	0.118	0.442	0.820	0.583	0.197	0.776	0.268
	Accuracy %	99.0	99.0	99.2	93.1	91.1	97.7	99.0
(**b**)								
		**Total**	**Buccolingual**	**Mesiodistal**	**Vertical**	**Inclination**	**Angulation**	**Rotation**
		**L**	**LY**	**LX**	**LZ**	**Theta (Y)**	**Phi (X)**	**Psi (Z)**
34,44	T0–TS	2.15 ± 1.1	0.97 ± 0.7	1.62 ± 1.05	0.9 ± 0.24	4.01 ± 2.24	5.56 ± 3	7.12 ± 2.45
	T0–T1	2.32 ± 1.32	0.96 ± 0.84	1.53 ± 1.09	0.84 ± 0.31	4.31 ± 2.53	5.13 ± 2.84	6.53 ± 2.36
	*p*	0.336	0.890	0.549	0.631	0.613	0.498	0.141
	Accuracy %	99.0	99.0	94.4	93.3	99.0	92.3	91.7
35,45	T0–TS	2.28 ± 1.02	0.93 ± 0.59	1.66 ± 0.9	0.99 ± 0.38	4.54 ± 1.71	6.45 ± 2.99	5.11 ± 3.46
	T0–T1	2.25 ± 1.14	0.97 ± 0.93	1.46 ± 1	0.86 ± 0.37	3.78 ± 1.9	6.26 ± 2.47	5.27 ± 3.87
	*p*	0.898	0.793	0.199	0.406	0.004 *	0.650	0.793
	Accuracy %	98.7	99.0	88.0	86.9	83.3 *	97.1	99.0
36,46	T0–TS	2.14 ± 0.84	0.89 ± 0.43	1.35 ± 1.07	1.14 ± 0.58	4.08 ± 2	4.57 ± 2.47	4.18 ± 1.41
	T0–T1	1.94 ± 0.98	0.86 ± 0.51	1.31 ± 1.06	0.86 ± 0.5	5 ± 2.12	4.23 ± 1.97	3.68 ± 2.03
	*p*	0.145	0.628	0.723	0.005 *	0.051	0.389	0.275
	Accuracy %	90.7	96.6	97.0	75.4 *	99.0	92.6	88.0
37,47	T0–TS	2.38 ± 0.74	1.19 ± 0.47	1.69 ± 1.11	0.9 ± 0.51	4.8 ± 2.04	6.06 ± 3.46	6.81 ± 2.01
	T0–T1	2.25 ± 0.88	1 ± 0.69	1.57 ± 1.32	0.81 ± 0.57	5.2 ± 2.6	5.15 ± 2.6	6.17 ± 2.07
	*p*	0.441	0.297	0.482	0.442	0.273	0.202	0.179
	Accuracy %	94.5	84.0	92.9	90.0	99.0	85.0	90.6

* *p* < 0.05 indicates statistically significant difference between predicted (T0–TS) and achieved (T0–T1) movements.

**Figure 9:**
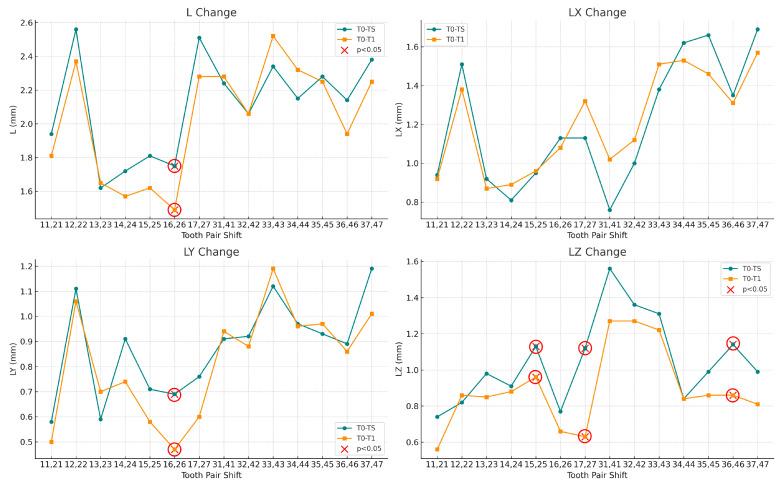
Linear tooth movement measurements comparing planned (T0–TS, green) and achieved (T0–T1, orange) displacements. L: total displacement; LX: buccolingual (anterior teeth)/mesiodistal (posterior teeth); LY: mesiodistal (anterior teeth)/buccolingual (posterior teeth); LZ: vertical displacement. Red X markers (circled in red) indicate statistically significant differences (*p* < 0.05).

**Figure 10:**
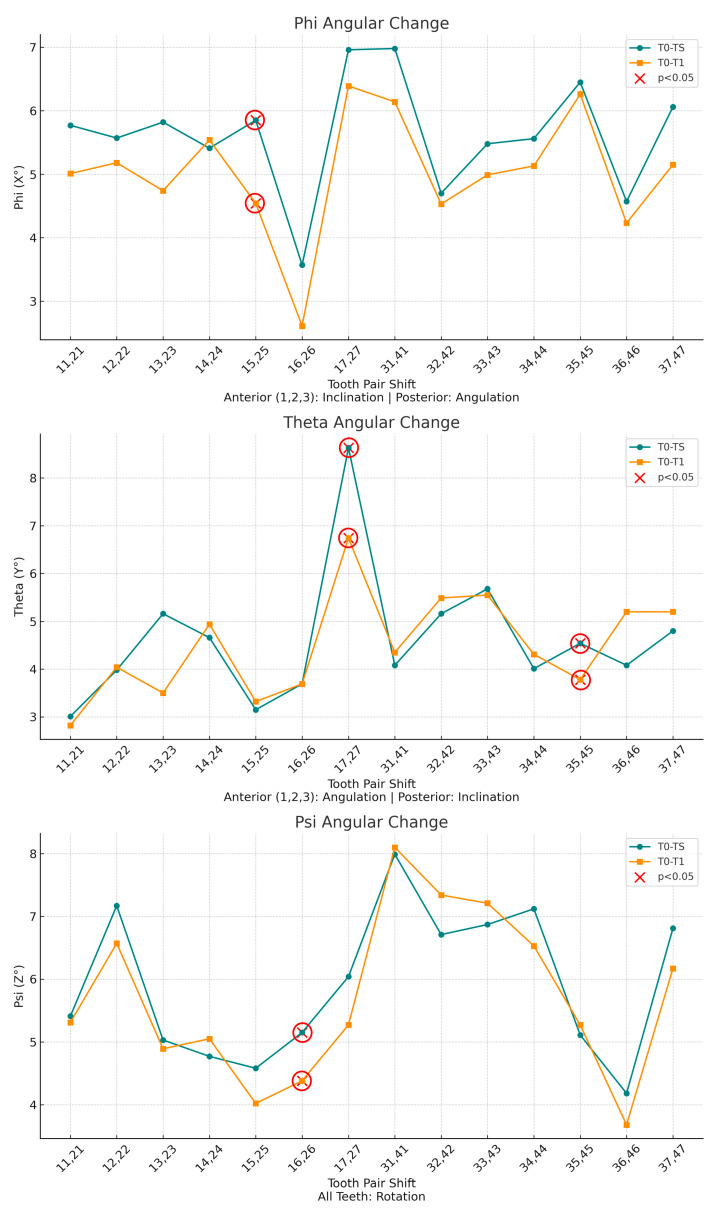
Angular tooth movement measurements comparing planned (T0–TS, green) and achieved (T0–T1, orange) rotations. Phi (X°): inclination (anterior teeth)/angulation (posterior teeth); Theta (Y°): angulation (anterior teeth)/inclination (posterior teeth); Psi (Z°): rotation around the tooth’s long axis. Red X markers (circled in red) indicate statistically significant differences (*p* < 0.05).

The distribution of linear and angular tooth movements across symmetrical tooth pairs, along with the mean values for each pair, is illustrated in Figure 9 and Figure 10.

### 3.2. Statistical Significance and Movement Precision

Ten parameters showed statistically significant differences between planned and achieved values (*p* < 0.05), primarily involving posterior teeth. In the maxillary posterior region, the largest deviations were observed for vertical displacement of teeth 17 and 27 (accuracy: 56.3%), buccolingual displacement of teeth 16 and 26 (68.1%), and angulation of teeth 15 and 25 (77.6%). In the mandible, fewer parameters reached statistical significance, with deviations observed for inclination of teeth 35 and 45 (83.3%) and vertical displacement of teeth 36 and 46 (75.4%). No statistically significant deviations were detected for mandibular anterior teeth. Maxillary anterior teeth demonstrated consistently high accuracy across most parameters.

### 3.3. Regional Movement Analysis

The fully customized self-ligating lingual appliance achieved an overall dentition accuracy of 92.1%. Mean accuracy for linear tooth movements was 94.5% ± 2.1% in the maxilla and 93.8% ± 2.5% in the mandible. The highest accuracy values were observed in the mandibular anterior region. In the maxillary anterior region, angulation accuracy was 97.2% and rotation accuracy was 95.7%, whereas vertical displacement accuracy was lower (87.1%). Posterior accuracy was lower overall, particularly in the maxillary arch (buccolingual: 77.5%; vertical: 80.9%). In the mandible, posterior accuracy remained higher (total displacement: 95.7%), despite reduced vertical displacement accuracy (86.4%).

## 4. Discussion

### 4.1. Principal Findings and Clinical Implications

The higher predictability observed in mandibular anterior teeth may be attributed to biomechanical characteristics of lingual orthodontics. Bracket positioning closer to the center of resistance, combined with simpler root morphology, facilitates more controlled force application and reduces the risk of uncontrolled tipping. Clinically, these findings indicate that customized self-ligating lingual appliances are well suited for anterior alignment. Except for a single deviation observed in tooth 36 for vertical displacement (LZ), mandibular teeth generally showed good agreement between predicted and achieved positions.

In contrast, greater deviations were observed in the maxillary posterior region, particularly in teeth 16 and 17. These findings likely reflect limitations in vertical control and buccolingual molar movement with lingual mechanics. In addition to biomechanical factors, the more complex root morphology of posterior teeth may contribute to the greater deviations observed in these regions, representing a challenge for precise posterior tooth movement with lingual orthodontic systems. Although linear accuracy remained acceptable, angular control, especially torque expression, was less predictable. Clinically, this underscores the importance of realistic treatment planning, the selective use of auxiliary mechanics, and conservative objectives when substantial molar repositioning is required.

### 4.2. Comparison with Previous Studies

The present findings are consistent with previous studies reporting higher accuracy in anterior regions and reduced predictability in posterior teeth. Pauls et al. reported translational deviations below 0.5 mm and angular discrepancies under 4.6°, particularly in incisors, a finding that is in line with the high anterior accuracy observed in the present study [2]. In a larger sample, Grauer and Proffit evaluated Incognito (3M Unitek, Monrovia, CA, USA) cases and found accuracy values generally below 1 mm and 4°, with the exception of second molars, a result that is consistent with the observation that posterior teeth present greater challenges in vertical and angular control [28]. Sharp et al. assessed the Incognito Lite system, a partial-arch appliance focused primarily on anterior correction rather than full-arch mechanics. Their results showed that while angular (tip and torque) and rotational movements were consistently achieved within ±3°, translational discrepancies were more pronounced, with average deviations reaching 1.0 mm in maxillary central incisors [34]. Fernandes et al. compared Incognito with iLingual 3D (GAC International, Bohemia, NY, USA) and Lingual Matrix (Adenta GmbH, Gilching, Germany), showing that Incognito achieved the highest accuracy in all parameters except in-out positioning, while all three systems proved clinically reliable for anterior tooth movements [24].

For non-customized lingual systems, Albertini et al. demonstrated that the lingual straight-wire technique achieved 84–92% accuracy for torque, tip, and rotation in incisors, canines, and premolars, whereas accuracy was lower in molars (52–81%) [26]. Scisciola et al. reported that passive self-ligating lingual straight-wire appliances with square slots achieved mean accuracy values of 77.25% for torque, 78.41% for tip, and 77.99% for rotation, with accuracy decreasing from anterior to posterior regions [25]. In parallel, Palone et al. showed that the Suresmile^®^ (Dentsply Sirona, Charlotte, NC, USA) lingual technique demonstrated even lower accuracy values of 60.11% for torque, 53.52% for tip, and 59.19% for rotation, highlighting the need for overcorrections during orthodontic planning [27]. These studies relied on prefabricated rather than fully customized lingual brackets, which may partly explain their lower accuracy compared with CAD/CAM-based fully customized systems.

Clear aligner therapy has been widely evaluated with respect to the predictability of complex tooth movements, providing a useful point of comparison with fixed appliance systems. Kravitz et al. reported a mean overall accuracy of 41%, identifying extrusion and rotation as the least predictable movements [33], while Haouili et al. observed a mean accuracy of approximately 50% for Invisalign^®^ (Align Technology, San Jose, CA, USA), with crown tip showing the highest predictability (56%) and rotation the lowest (46%) [22]. More recently, Migliorati et al. reported improved accuracy values for 3D-printed aligners, with 67.6% for torque, 64.2% for tip, and 72.0% for rotation, and particularly high predictability for transverse movements (99.6%) [16]. Further investigations have shown that limitations persist for larger or more demanding movements. D’Antò et al. demonstrated that even after 15 aligners, extensive mesiodistal and vertical corrections exhibited notable deviations, especially in molars [19]. Similarly, Li et al. reported that aligner software tends to overestimate achievable tooth movement in staged treatments, particularly for posterior displacement [15]. Although Bilello et al. described more favorable outcomes for Invisalign^®^, especially in mandibular central incisors, reduced predictability remained evident for movements of greater magnitude [23]. In the present study, angular changes exceeding 6° of inclination and vertical displacements greater than 2 mm were frequently planned, particularly in posterior teeth, creating a more demanding biomechanical scenario and providing a rigorous test of the performance of the fully customized self-ligating lingual appliance.

### 4.3. Limitations and Future Directions

The findings of this study should be interpreted considering limitations. Although sample adequacy was confirmed, planned movements were generally moderate and did not encompass a wide range of malocclusion types. The retrospective design limited treatment standardization, and minor digitization and landmarking variability may have influenced superimposition accuracy despite validated protocols. Factors such as wire–bracket play, torque expression limitations, and archwire coordination may partially explain reduced accuracy in certain parameters. As only one fully customized lingual system was evaluated, the findings may not be directly generalizable to other systems. Although contralateral tooth pairing reduced intra-subject variability, tooth movement within an arch is biomechanically interdependent, representing an inherent limitation of this analytical approach [35,36].

Despite these limitations, the study provides a detailed three-dimensional evaluation of a fully customized self-ligating lingual appliance and contributes meaningful clinical insight. Future prospective, multi-center studies with larger and more heterogeneous samples are needed, and emerging artificial intelligence–based tools may further enhance the objective assessment of orthodontic treatment accuracy [37].

## 5. Conclusions

The fully customized self-ligating lingual orthodontic appliance showed a high level of accuracy in achieving planned tooth movements in patients with mild to moderate malocclusions. Accuracy was generally higher in anterior teeth, while posterior regions showed reduced predictability, particularly for vertical and angular movements. Future multi-center studies including more complex malocclusion types are needed to further assess the clinical performance of this system.

## Figures and Tables

**Table 1:** Parameters used for tooth movement analysis, including angular (Phi, Theta, Psi) and linear (L, LX, LY, LZ) measurements with axis-specific interpretations for anterior and posterior teeth.

Parameter	Axis	Type	Definition/Direction
Phi (X°)	X	Angular	Inclination (anterior teeth)/Angulation (posterior teeth)
Theta (Y°)	Y	Angular	Angulation (anterior teeth)/Inclination (posterior teeth)
Psi (Z°)	Z	Angular	Rotation around tooth’s long axis
L	3D vector (XYZ)	Linear	Total displacement calculated as the vector magnitude of LX, LY, and LZ
LX	X	Linear	Buccolingual (anterior teeth)/Mesiodistal (posterior teeth)
LY	Y	Linear	Mesiodistal (anterior teeth)/Buccolingual (posterior teeth)
LZ	Z	Linear	Vertical displacement (intrusion/extrusion)

## Data Availability

The data presented in this study are available on reasonable request from the corresponding author. The data are not publicly available due to privacy and ethical restrictions related to patient confidentiality.

## Abbreviations

The following abbreviations are used in this manuscript:

3D	Three-Dimensional
ARS	Air Rotor Stripping
CAD/CAM	Computer Aided Design/Computer Aided Manufacturing
FMA	Frankfort Mandibular Plane Angle
NiTi	Nickel Titanium
SL	Self-Ligating
SPSS	Statistical Package for the Social Sciences
STL	Standard Tessellation Language
TMMDA	Turkish Medicines and Medical Devices Agency
